# mtFRC: depth-dependent resolution quantification of image features in 3D fluorescence microscopy

**DOI:** 10.1093/bioadv/vbad182

**Published:** 2023-12-18

**Authors:** Neil Wright, Christopher J Rowlands

**Affiliations:** Department of Bioengineering, Imperial College London, London SW7 2AZ, United Kingdom; Department of Bioengineering, Imperial College London, London SW7 2AZ, United Kingdom

## Abstract

**Motivation:**

Quantifying lateral resolution as a function of depth is important in the design of 3D microscopy experiments. However, for many specimens, resolution is non-uniform within the same optical plane because of factors such as tissue variability and differential light scattering. This precludes application of a simple resolution metric to the image as a whole. In such cases, it can be desirable to analyse resolution only within specific, well-defined features.

**Results:**

An algorithm and software are presented to characterize resolution as a function of depth in features of arbitrary shape in 3D samples. The tool can be used to achieve an objective comparison between different preparation methods, imaging parameters, and optical systems. It can also inform the design of experiments requiring resolution of structures at a specific scale. The method is demonstrated by quantifying the improvement in resolution of two-photon microscopy over confocal in the central brain of *Drosophila melanogaster*. Measurement of image quality increases by tuning a single parameter, laser power, is also shown. An ImageJ plugin implementation is provided for ease of use via a simple Graphical User Interface, with outputs in table, graph, and colourmap formats.

**Availability and implementation:**

Software and source code are available at https://www.imperial.ac.uk/rowlands-lab/resources/.

## 1 Introduction

Quantifying image quality is important for both experiment design and the development of optical instruments. In biology, it may be necessary to resolve structures at a certain level of detail; however, features which can be readily observed in one part of a sample may be unresolvable elsewhere. Similarly, having an objective metric of quality in microscopy allows comparison and improvement of instruments under various adverse imaging conditions, as opposed to the favourable conditions that occur near a tissue surface. Since the final quality is a function of both the sample and optical system as a whole, this should be reflected in any metric.

In 3D samples, image quality is also highly dependent on tissue depth, with quality degrading due to light attenuation and distortion caused by scattering. Previous approaches to quantify this effect have sometimes relied on signal intensity (Dong *et al.* 2000, Balu *et al.* 2009) as a metric. However, intensity lacks an obvious practical interpretation and may not always correlate with image quality. Another approach is to use a score based on arbitrary units (Preusser *et al.* 2021), though this also has the same issue of interpretability. In theory, using resolution as a metric directly can solve these issues.

Resolution refers to the minimum distance at which two separate objects are distinguishable. Mathematically, this can be defined based on either spatial frequency contrast or distance. The modulation transfer function (MTF) can be used to characterize the former, while the Rayleigh Criterion is an example of the latter. This states that two Airy discs are resolvable if the centre of one disc lies within or outside the first minimum of the diffraction pattern of the other (Rayleigh 1879).

While specially manufactured test samples can be used to assess the performance of a system using either method, natural images are unlikely to contain patterns of known contrast or distance that can be used directly to measure resolution. Estimation methods must therefore be used instead. For lateral resolution, this can be done by either estimating the MTF (Rowlands *et al.* 2015) or using an approach based on Fourier ring correlation (FRC) (Banterle *et al.* 2013). The latter technique originates in electron microscopy and involves finding the highest spatial frequency in an image distinguishable from noise (Saxton and Baumeister 1982).

However, a problem arises when applying a single measurement to more complex samples where resolution may be non-uniform across the image. This is often the case in 3D biological specimens where variations in factors such as tissue depth, type, or fluorophore concentration may create large differences in quality within the same optical plane.

Previously, it has been shown that FRC can be used to analyse local resolution by splitting the image into tiles (Culley *et al.* 2018), an approach recently used to analyse fine features in super resolution images (Zhao *et al.* 2023). Here, applying this approach to 3D fluorescence microscopy, we develop an algorithm to isolate features of arbitrary 3D morphology within a sample and quantify resolution within those features as a function of depth.

The method is demonstrated by analysing the central region of the dissected brain of *Drosophila melanogaster*, a model organism widely used in neurobiology. Due to its 3D morphology, optical sections of the fly brain can contain regions of heterogeneous resolution, with particular differences between the central brain and optic lobes caused by differing levels of light scattering. By calculating the mean FRC value within the central brain only, we show how to characterize resolution within any arbitrarily shaped region of an image, and quantify its degradation with increasing depth.

Results comparing confocal and two-photon imaging are presented. These align with previous findings on the highly scattering nature of the fly brain compared to mammalian brain, even when two-photon microscopy is used (Hsu *et al.* 2019). Additionally, we show how the tool can be used to quantify improvements in resolution when tuning a single parameter, laser power. Finally, the performance of FRC to estimate resolution in 3D stacks is evaluated by comparing its results to full width at half maximum (FWHM) measurements of sub-diffraction size fluorescent beads.

The software is generally suitable for analysing resolution in features of 3D samples where image quality is non-uniform. An open-source ImageJ plugin implementation is provided, allowing simple use via a Graphical User Interface. Results can be generated in table, graph, and resolution colourmap formats. Since the output metric is based on resolution, values have a practical interpretation which can be used to inform experimental considerations, as well as objectively compare results from different sample preparations, imaging parameters, and optical systems.

## 2 Methods

### 2.1 Sample preparation

#### 2.1.1 Green fluorescent protein *Drosophila* brain

Green Fluorescent Protein (GFP) was expressed pan-neuronally in *Drosophila* by crossing nSyb-GAL4 [Bloomington Drosophila Stock Center (BDSC) #68222] with 10XUAS-IVS-mCD8::GFP (BDSC #32187). Adult male and female flies were anaesthetized by low temperature and brains dissected in phosphate-buffered saline (PBS) using fine forceps (Dumont #55 and #5SF) (Wu and Luo 2006). Dissected brains were transferred to a PBS-filled glass-bottom 35 mm confocal dish (VWR) coated with poly-d-lysine (∼100 µg/ml; Sigma) and oriented dorsal side down.

#### 2.1.2 Fixed *Drosophila* brain

Fixation and phalloidin staining were based on the method described in Behnke *et al.* (2021). Briefly, a fly brain was dissected as for GFP brains and transferred to an Eppendorf tube containing 4% paraformaldehyde in PBS and 0.5% Tween-20 (PBST). The tube was placed on a rocker for 20 min. Following fixation, the brain was washed three times with PBST, with 20 min on the rocker after each wash. Invitrogen ActinGreen 488 ReadyProbes (AlexaFluor 488 phalloidin) reagent was added at a concentration of two drops per ml. The tube was placed back on the rocker for 16 h. The brain was then washed three times with PBST with 20 min on the rocker after each wash, and once with PBS with 30 min on the rocker. The brain was mounted on a SuperFrost Plus slide using ProLong Diamond Antifade Mountant (Invitrogen).

#### 2.1.3 Fluorescent beads

Fluorescent beads of diameter 210 nm (Invitrogen TetraSpeck Microspheres) were prepared in 1% agarose (Thermo Scientific). The sample was transferred to a confocal dish for imaging.

### 2.2 Confocal and two-photon microscopy

Confocal and two-photon (2p) images were both taken using the same commercial Leica SP5 inverted microscope at the Facility for Imaging by Light Microscopy (FILM) at Imperial College London.

For all images, a 20× 0.7 NA dry objective (Leica) was used for imaging and a photomultiplier tube was used to detect fluorescence. The bit depth was set to 16 bits and line scan rate set to 400 Hz. For fly brains, the pixel size was 94.6 nm for GFP stacks used for ‘single image’ analysis and 189 nm for phalloidin stacks used for ‘two image’ analysis; the step size for both was 10 µm. For fluorescent beads, the pixel size was 93.1 nm and step size was 0.5 µm.

Confocal imaging used an Argon laser at 488 nm and pinhole size of 1 Airy Unit. Two-photon imaging used a Mai Tai DeepSee laser (Newport Spectra-Physics) tuned to 920 nm and the pinhole fully opened.

Each fly brain was imaged multiple times using different modalities. Because initial results found photobleaching to be minimal except for higher power 2p stacks, the order was not randomized and 2p stacks were always captured last.

Power was measured using a Thorlabs S170C power sensor for the Argon laser, and S425C for the DeepSee laser.

### 2.3 Image analysis

#### 2.3.1 Algorithm

**Figure 1.**
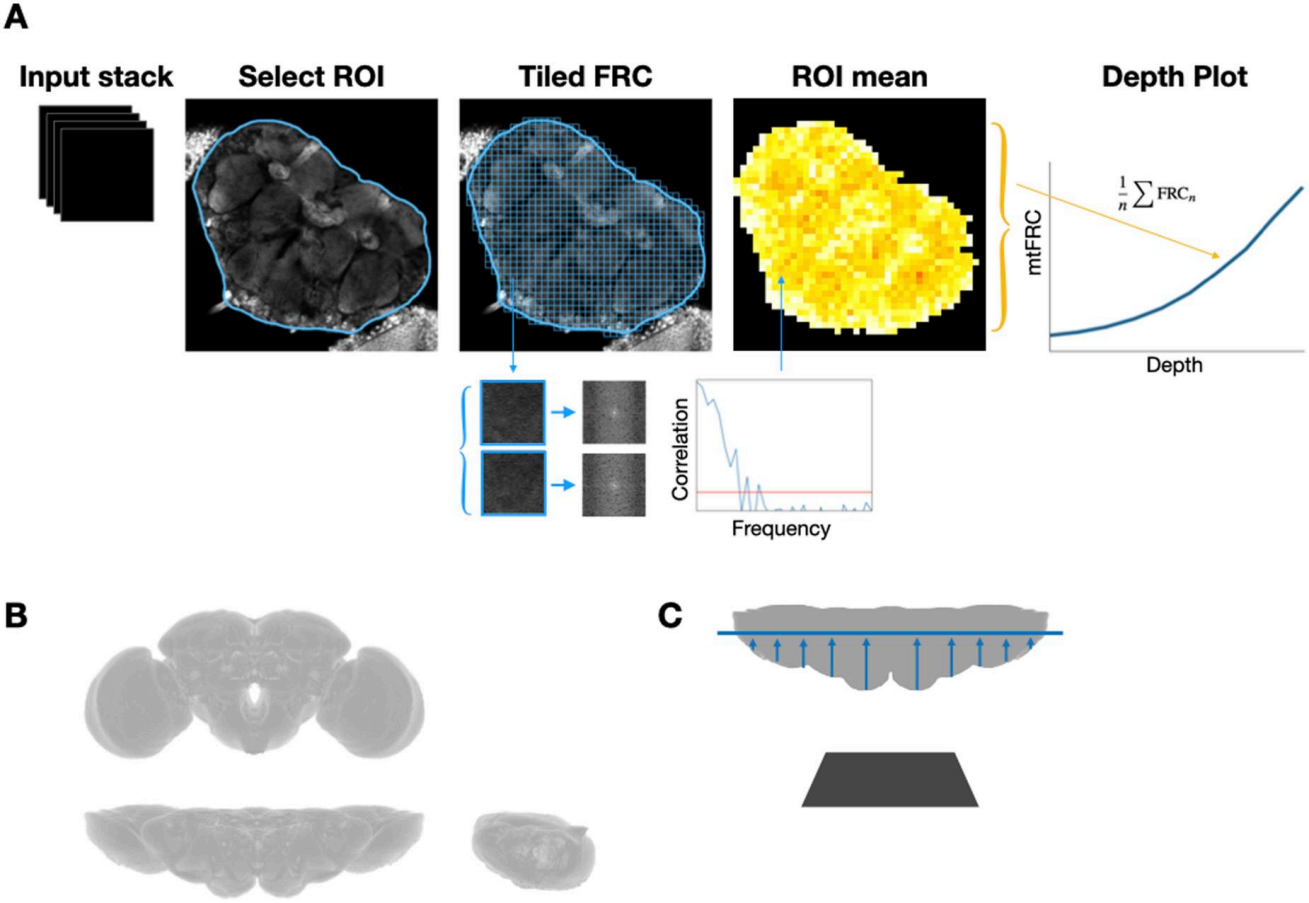
(A) Overview of the method to calculate mtFRC. First, an ROI is drawn over the feature to be analysed in each image in the Z-stack. The image is then divided into small tiles, and the FRC value for each tile within the ROI is calculated by selecting the highest spatial frequency whose correlation coefficient is greater than the cut-off value. Finally, the mean value for each ROI is computed and plotted as a function of depth. Since FRC represents the minimum resolvable distance, lower values correspond to better image quality. (B) Top, Front, and Side views of the *Drosophila* brain, showing the 3D structure. (C) *Drosophila* brain with the dorsal side facing down towards the microscope objective (dark grey), as imaged in this study. The horizontal line represents an optical plane. Variable tissue depths (arrows) result in differing levels of light scattering, contributing to resolution heterogeneity within the image. *Drosophila* brain images in B and C based on graphics from virtualflybrain.org (Court *et al.* 2023).

The algorithm to compute the mean tiled FRC (mtFRC) in a region of interest (ROI) of arbitrary shape is illustrated in Fig. 1. First, ROIs are drawn manually for each image in the Z-stack. For the data used here, the central brain was the feature of interest. Each image is then split into small (e.g. 64 × 64 pixels) tiles. For each tile in the ROI, FRC is calculated. In general, calculating FRC requires two independent images. Both ‘single image’ and ‘two image’ versions of FRC are implemented. For the ‘single image’ version, each full image is first split into four sub-images as described in Koho *et al.* (2019). The final FRC value is the average result from two sub-image pairs. FRC is calculated according to the standard formula of computing the Pearson correlation coefficient of rings of increasing radius in the Fourier transforms of the two images according to Equation (1):

(1) $$\text{FRC}(r)=\frac{\sum_{i\in R}F_{1}(r_{i})\cdot F_{2}(r_{i})*}{\sqrt{\sum_{i\in R}|F_{1}(r_{i}){|}^{2}\cdot \sum_{i\in R}|F_{2}(r_{i}){|}^{2}}},$$

where $F_{1}$ is the Fourier transform of the first image and $F_{2}*$ is the complex conjugate of the Fourier transform of the second image; the numerator is a real number (Van Heel 1987). The inverse of the frequency below which the correlation drops below a fixed 1/7 cut-off is the FRC value (Nieuwenhuizen *et al.* 2013).

For each depth, mtFRC is taken to be the mean FRC value of all tiles in the ROI for that depth. The output graph is generated by plotting mtFRC as a function of depth for the stack as a whole. The ImageJ plugin implementation can output results in table, graph, and resolution colourmap formats based on user selections. All code is written in Java.

#### 2.3.2 High-detail colourmaps

**Figure 2.**
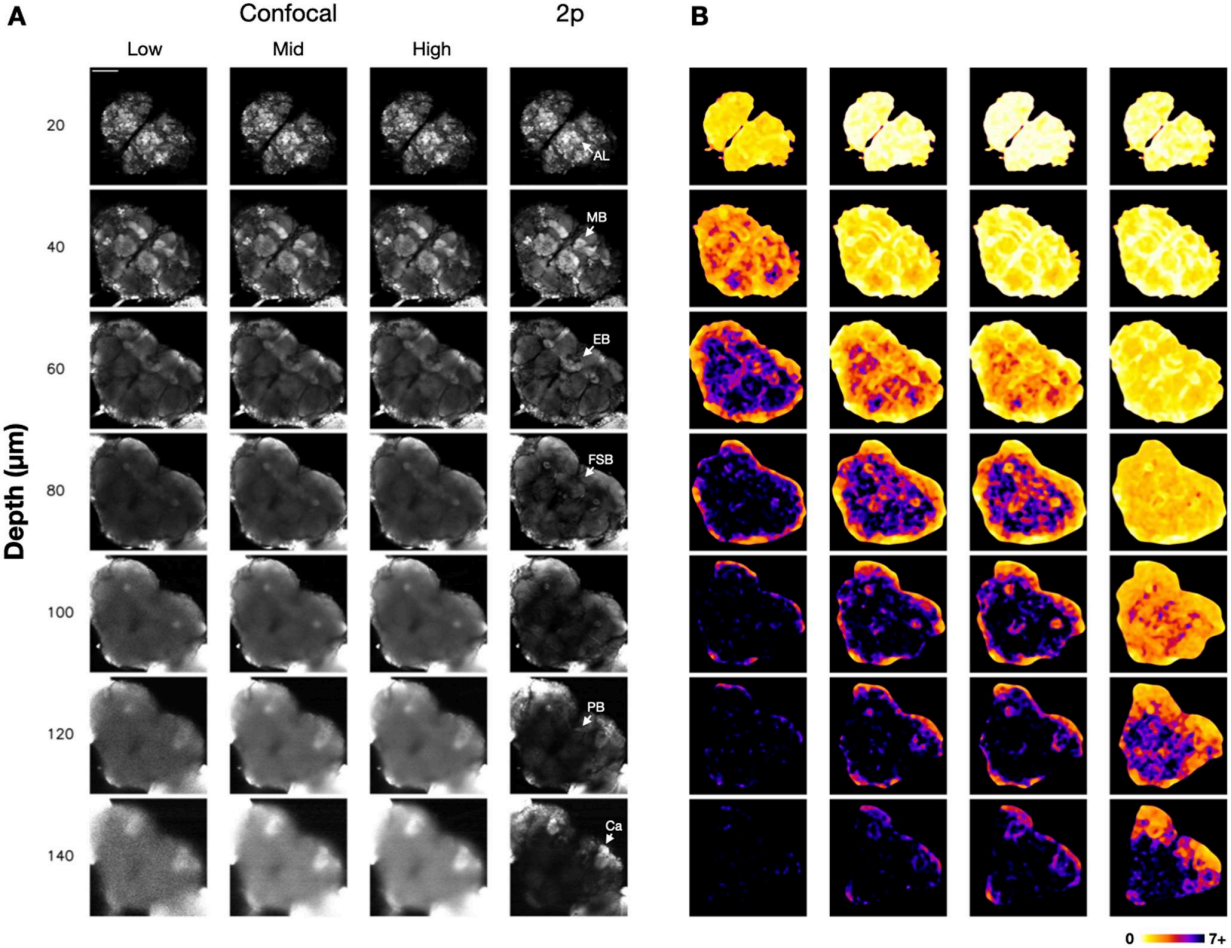
(A) Example images of an nSyb>GFP brain at various depths for each imaging modality. Confocal power levels were 9 µW (low), 100 µW (mid), and 224 µW (high). Two-photon power was 22 mW. Each image has been individually optimized for brightness and contrast to highlight image quality. Brain structures are indicated by arrows. Scale bar = 100 µm. (B) High-detail rolling FRC colourmaps of the central brain ROI, generated from the same stacks shown in A. The scale represents minimum resolvable distance (FRC) from 0 to 7+ µm. AL, antennal lobes; EB, ellipsoid body; FSB, fan-shaped body; PB, protocerebral bridge; Ca, mushroom body calyx.

High-detail FRC colourmaps (Zhao *et al.* 2023) were also generated to highlight precise variations in resolution across images (Fig. 2). The approach to generate these colourmaps was similar to the method described above, except that, instead of dividing the image into tiles, a 64 × 64 pixel block centred on each pixel was scanned across the ROI. The FRC value for each block was converted to a colour value and used to draw a single pixel at the corresponding coordinates in the colourmap. Artefacts were smoothed by applying a Median Filter (Radius = 10) using Fiji (Schindelin *et al.* 2012).

#### 2.3.3 Fluorescent bead resolution

The confocal stack of fluorescent beads in agarose was processed by custom Java code to extract small substacks containing each individual bead. The centre of each bead was determined by applying a Median Filter of radius 1 pixel to the substack, and finding the maximum intensity pixel (MIP). The FWHM for *X* and *Y* was determined by fitting a Gaussian curve to horizontal and vertical profile lines respectively through the MIP. The FWHM is calculated from the standard deviation by the relation:

(2) $$\text{FWHM}=2\sqrt{2\ln2}\sigma .$$

The final FWHM value is the average of *X* and *Y* FWHM values.

FRC was calculated for each bead by extracting a single 64 × 64 pixel tile centred on the MIP and applying ‘single image’ FRC using our plugin; this corresponded to a sub-image size of 32 × 32 pixels. This smaller tile size was chosen to minimize bead overlap and reduce the amount of background in each image.

## 3 Results and discussion

### 3.1 Theoretical considerations for imaging parameters

To determine the pixel size required to capture all information within the image, Abbe’s diffraction formula was used to estimate the minimum theoretical distance resolvable by the system (Rayleigh 1879):

(3) d=λ/(2NA).

Here, *d* = 349 nm for confocal and 657 nm for 2p imaging. Shannon–Nyquist sampling requires using half these values (Shannon 1949). Therefore, a pixel size of 189 nm was chosen, close to the value required.

Since FRC requires two independent images, we used both conventional ‘two image’ FRC and ‘single image’ FRC (Koho *et al.* 2019), whereby a single image is split into sub-images. Single image FRC may be more convenient and has the advantage that Z alignment is ensured. In contrast, if two stacks are captured sequentially, subtle shifts in Z position can occur occasionally, compromising results; capturing both images for each depth before adjusting the Z value should prevent this issue. To achieve equivalent sampling for single and two image analysis, single image stacks were captured with a pixel size of 94.6 nm, equivalent to a pixel size of 189 nm for each sub-image.

Three power levels were used for confocal stacks. A power of 100 µW was used as the ‘middle’ setting based on tuning the power to the level required to adequately visualize layers close to the surface. To illustrate the effects of power adjustments, a ‘low’ level of ∼10% of this value (9 µW), and a ‘high’ level of approximately twice this value (224 µW), were also used. The 2p power level was 22 mW, close to the value of around 20 mW commonly used to image the fly brain (Hsu *et al.* 2019).

### 3.2 Analysis of image stacks with non-uniform resolution

Initial attempts to characterize image quality as a function of depth found that using a single FRC calculation for each full image in the stack led to inconsistent and non-monotonic results. FRC colourmaps (Zhao *et al.* 2023) revealed that this was due to non-uniformity of resolution within images, particularly the contrast between the central brain and optic lobes, which begin at deeper optical planes (Fig. 1B and C) and thus appear as higher resolution areas due to decreased light scattering. Apart from the optic lobes, the effect of differential light scattering on resolution is also apparent at the edges of the central brain, where tissue is thinnest and image quality better, and in the middle of the brain, where tissue is thickest and image quality worst (Fig. 2). The colourmaps also indicated that the outlines of major brain structures, such as the antennal lobes, mushroom body, and central complex, appeared as slightly higher resolution features, likely due to the presence of sharp ‘edges’ resulting from differing GFP concentrations (Fig. 2). However, for *Drosophila* brain, light scattering appeared to be the major determinant of resolution, rather than factors such as tissue heterogeneity.

This raised the question of how a single metric could characterize image quality as a function of depth when quality varies within each plane. It was decided that a method with general practical utility would be a feature-based approach. In this methodology, the user selects an image feature where quality is relatively consistent—e.g. where tissue type and light scattering are similar—and resolution is then characterized only within this region. This approach has the practical advantage that it can inform experimental work where specific structures need to be resolved at a certain scale, because it can be restricted to any user-defined local area. In addition, it can also provide an objective comparison of image quality between different sample preparation methods and imaging modalities. For the *Drosophila* brain images used here, the central brain was an obvious choice of feature because the tissue type was homogeneous and levels of light scattering had relatively little variation throughout.

The method used to isolate image features requires certain considerations. Simply cropping the image has the disadvantage that only rectangular areas can be analysed, rather than the irregular shapes which are common in natural images. An alternative approach is to mask out other features. However, this risks creating artificially sharp edges in the image which may distort FRC-based estimation of resolution. To overcome these issues, the mtFRC method was developed based on splitting the image into small tiles, and selecting only the tiles contained within the feature of interest. Calculating the mean of these tiles then accounts for small variations in resolution within the feature, thereby providing a more robust overall metric. This approach allows characterization of resolution in ROIs of arbitrary shape, providing results which remain relatively consistent across samples. Our implementation of FRC used the widely used cut-off value of 1/7 (Nieuwenhuizen *et al.* 2013). However, we note that the optimal cut-off value is a matter of debate in the literature and alternative approaches exist, such as σ-factor and bit-based curves (van Heel and Schatz 2005).

If more fine-grained resolution information is required, a ‘rolling block’ approach can be applied in a similar way to the tiling method, though this comes at the cost of greatly increased computation. We found that the rolling FRC method produced comparable results to the tiling method in terms of mean value within the ROI (Supplementary Fig. S1), but at the cost of greatly increased computation. Therefore, the tiling method was used in our implementation. As a simple example, assuming a tile size of 64 × 64 pixels, a 1024 × 1024 pixel square ROI requires $16^{2}$ = 256 FRC calculations for the tiling method, but $1024^{2}$ = 1 048 576 FRC calculations for the rolling block method.

One limitation of the mtFRC method is that while use of small tiles provides highly localized information, tile size also constrains the lowest spatial frequency which can be correlated. This results in a trade-off between minimizing the area to which resolution information is localized and maximizing the range of resolutions which can be detected. A tile size of 64 × 64 pixels was found to provide a reasonable compromise in this regard, though larger sizes can be used when fine segmentation is not important.

In principle, the method could also be applied to camera-based microscopy images. Several considerations are noted in this regard. Firstly, achieving sufficient sampling for single-image FRC may be more challenging compared to laser scanning methods, because pixel sampling is fixed by the design of the microscope. To reduce the sampling requirements, conventional two-image FRC can be used to calculate mtFRC instead, a feature supported by our implementation. Secondly, in our experience, FRC works best when images are as free of artefacts as possible, because the FRC algorithm is unable to distinguish between object features and artefacts with high spatial frequency content. Imaging modalities where artefact patterns cannot be suppressed may therefore not be ideal for use with any FRC-based analysis method.

Our implementation of mtFRC is made available as an open-source ImageJ plugin (https://www.imperial.ac.uk/rowlands-lab/resources/), allowing easy use by non-expert users. Currently, an ImageJ FRC plugin exists but only operates on whole images and requires an image pair (Burri 2018). The NanoJ-SQUIRREL plugin (Culley *et al.* 2018) is able to run blockwise FRC, but also requires two input frames and does not implement the mtFRC method of depth analysis developed here. Our plugin works on single images in addition to image pairs, allows analysis of 3D image features based on an ROI set and can generate plots of lateral resolution as a function of depth directly from the plugin.

### 3.3 Confocal and two-photon resolution in *Drosophila* brain

**Figure 3.**
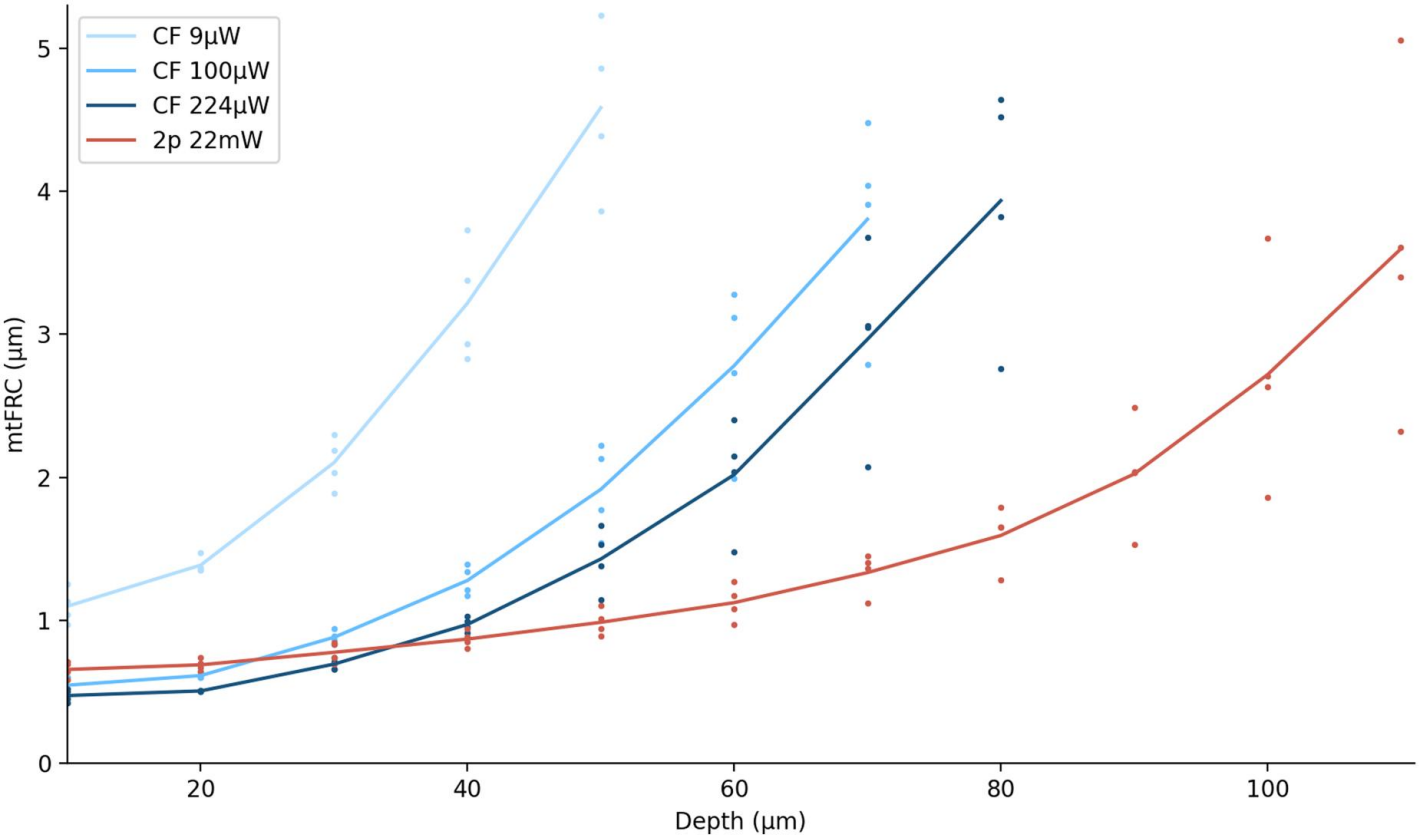
Resolution as a function of depth in the central region of the *Drosophila* brain for four different specimens of genotype nSyb>GFP. Values are plotted for depths where ≥95% of tiles contained correlating spatial frequencies for at least half the specimens. Three different confocal power levels (9, 100, and 224 µW) were compared with two-photon imaging (22 mW) using the mtFRC metric. Lines show means for each modality.

Two-photon (2p) imaging is widely used in neurobiology, including in *Drosophila* (Svoboda and Yasuda 2006). Use of higher-order excitation suppresses out of focus fluorescence, while longer wavelengths of light are less prone to scattering (Helmchen and Denk 2005). In the GFP brains here, the benefits of 2p (22 mW) were apparent for depths ∼35 µm and greater, with the difference increasing with depth (Fig. 3). For depths less than this, sufficiently powered confocal appeared to provide slightly sharper images, in line with the theoretical resolution benefits of shorter wavelengths (Rayleigh 1879). Despite the benefits of 2p, however, imaging depth was still limited in comparison to mammalian brain, where imaging up to 1.6 mm has been reported (Kobat *et al.* 2011). This aligns with a recent study on the optical properties of the fly brain, which attributed its highly scattering nature to extensive light scattering at the air–tissue interface in tracheae (Hsu *et al.* 2019). At a practical level, though deeper brain structures such as the mushroom body calyx and protocerebral bridge could be imaged using 2p imaging (Fig. 2), higher resolution imaging of these regions may require three-photon microscopy (Hsu *et al.* 2019). A further consideration is that 2p may cause considerably more photobleaching than confocal, which may be a factor in certain experiments, such as those involving long-term imaging. Additionally, while the results here were based on dissected brains, resolution may vary *in vivo*.

**Figure 4.**
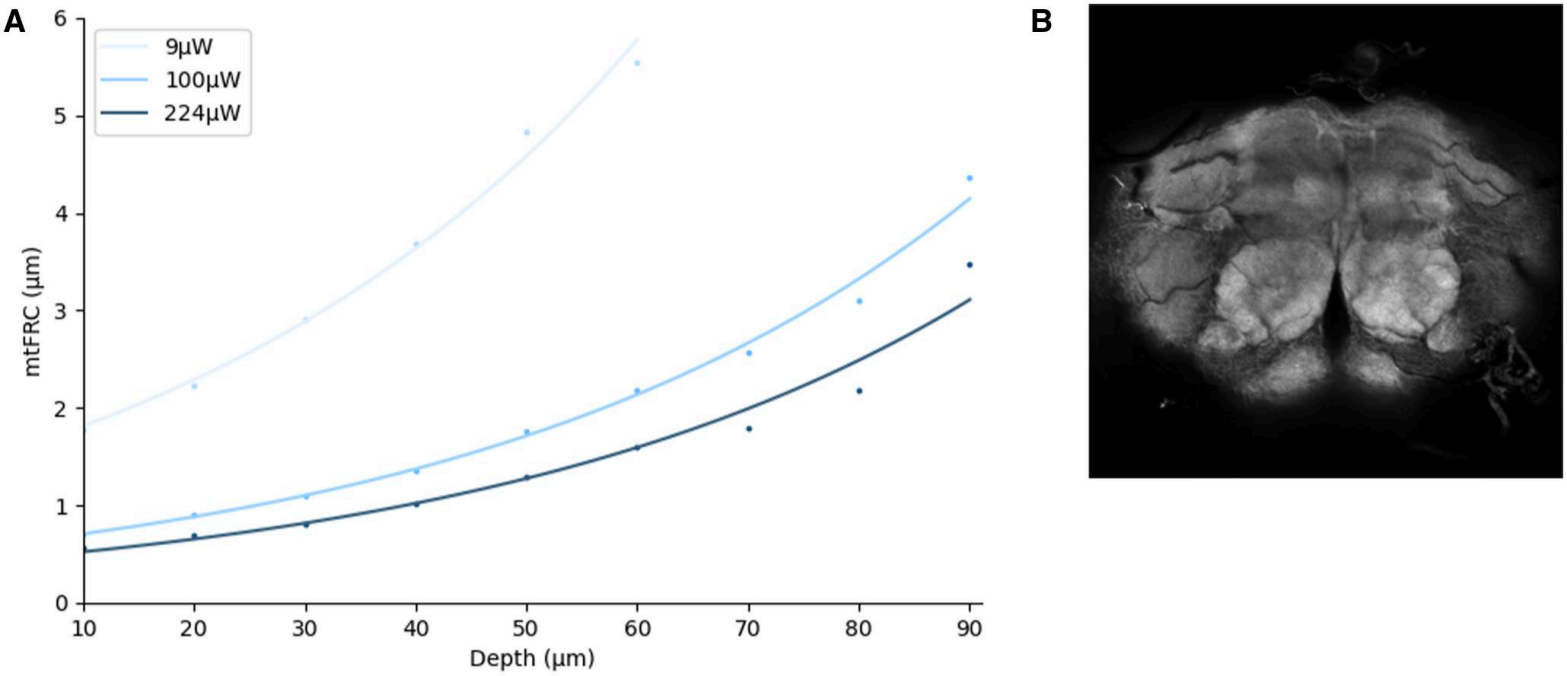
(A) Resolution as a function of depth in the central region of a fixed *Drosophila* brain stained with phalloidin, imaged using three confocal power levels (9, 100, and 224 µW). Values are plotted for depths where ≥95% of tiles contained correlating frequencies. Lines show exponential fit. (B) Example image from the stack.

Confocal laser power was used as an example to demonstrate how improvements in image quality through tuning a single parameter can be quantified using the method described (Fig. 3). In the results for GFP brains, each increase in power led to a measurable improvement in resolution, with the improvement becoming more pronounced with increasing depth. This can be explained by the general principle that, as the number of photons increases, signal increases linearly while noise increases by the square root of the number of photons, leading to a higher signal-to-noise ratio (SNR) (Sheppard *et al.* 2006). Imaging a sample with a different preparation method, fixation followed by phalloidin staining, showed the same effect (Fig. 4). Quantifying resolution in this way thus allows system tuning until requirements for a given experiment are met.

When normalized signal intensity is computed, there is an obvious distinction between linear and non-linear fluorescence methods, but less distinction between confocal imaging using different power levels, despite apparent differences in image quality when viewed by eye (Supplementary Fig. S2). In contrast, these differences are clearly reflected in terms of resolution via the mtFRC metric (Fig. 3).

### 3.4 Evaluation of FRC estimates using fluorescent beads

**Figure 5.**
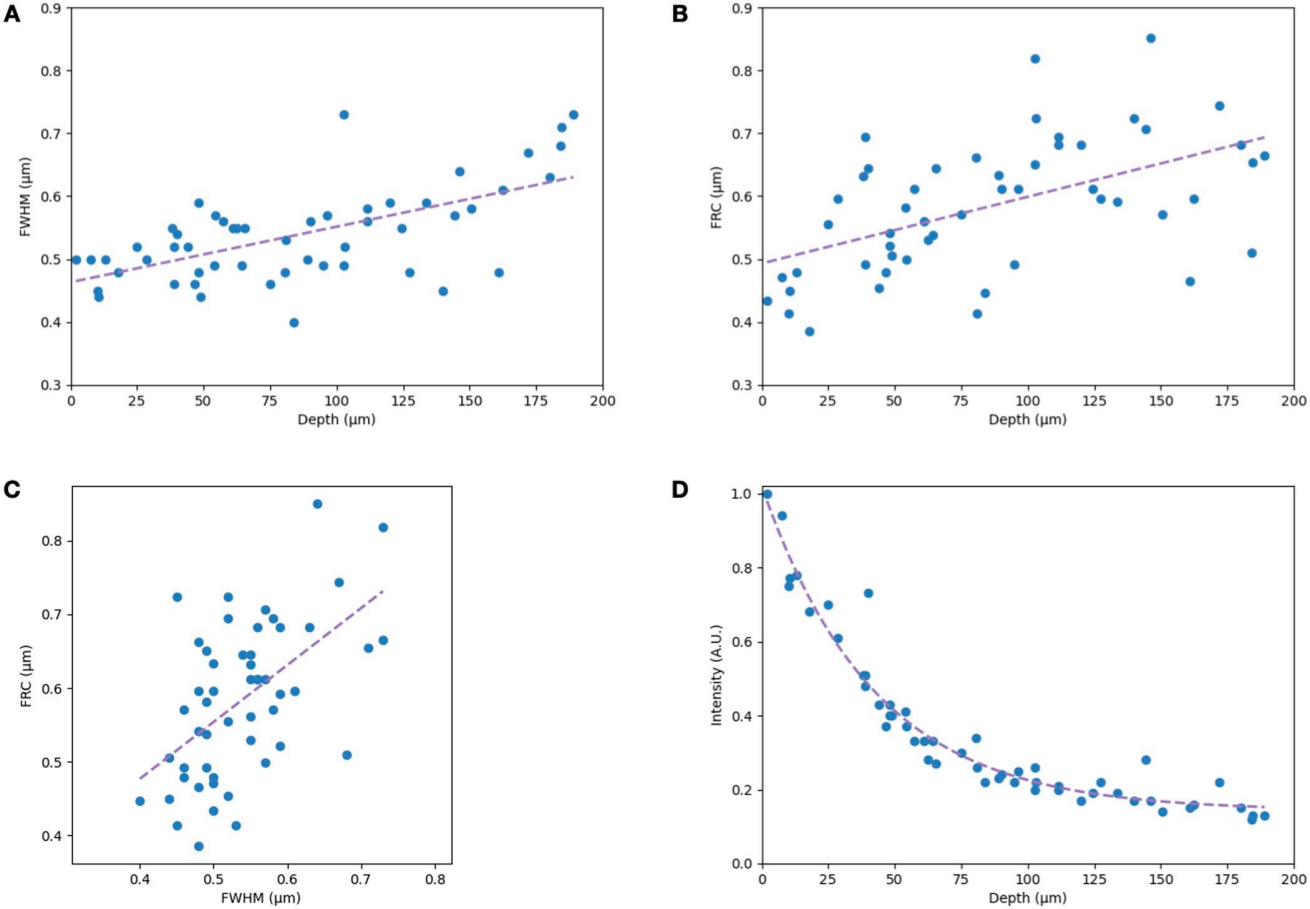
Comparison of FWHM and FRC of 210 nm fluorescent beads in 1% agarose imaged using confocal microscopy. (A) FWHM of beads as a function of depth. The line shows linear fit. (B) FRC of the same beads as a function of depth. Each FRC value is computed based on a 64 × 64 pixel tile containing the bead; these correspond to 32 × 32 pixel sub-image inputs to ‘single image’ FRC. The line shows linear fit. (C) FRC plotted versus FWHM, showing that both metrics provide comparable values for resolution. The line shows linear fit. (D) Normalized intensity of the maximum intensity pixel of each bead as a function of depth. The line shows exponential decay regression.

To evaluate how well FRC estimates resolution in 3D image stacks, we compared the FWHM with the FRC value of the point spread function of sub-diffraction size fluorescent beads in agarose (Fig. 5). Both metrics show a slight degradation of resolution with depth, likely due to spherical aberration (Egner and Hell 2006). Overall, FWHM and FRC were in relatively close alignment (mean difference = −5.8%, standard deviation = 15.0%), indicating that FRC provides real values for depth stacks. Moreover, at the sample surface where scattering is minimal, both FRC and FWHM results were similar to mtFRC estimates of confocal resolution at the surface of the fly brain (Figs 3 and 4).

It is noted that the gradient of resolution degradation appeared to be slightly sharper for FRC estimates compared to FWHM (Fig. 5A–C). We suggest this may be due to the contribution of background in the FRC calculations, though further investigation would be required to determine if this is a consistent trend. It is also noted that, while FWHM may be considered the ‘gold standard’ for resolution estimation, results are still variable depending on the software implementation used (Metz *et al.* 2023).

Finally, even though agarose is highly transparent to visible light, a large drop in intensity was observed in the bead sample (Fig. 5D). In contrast, the drop in resolution was relatively small when compared to the fly brain across the same depth range. This provides a further demonstration that signal intensity alone is an inadequate proxy for resolution.

## 4 Conclusion

The algorithm and software presented enable quantification of lateral resolution as a function of depth within any feature of arbitrary shape in 3D samples. This is achieved by splitting the ROI in each plane into small tiles and applying FRC to each tile, with the final value for each depth representing the mean value of tiles within the feature. The method was demonstrated by characterizing resolution at each depth in the central brain of *Drosophila* and quantifying the benefits of two-photon imaging over confocal. Measurement of image quality improvements through tuning a single parameter, laser power, was also shown in samples prepared with two different methods. In addition, the performance of FRC to estimate resolution in 3D stacks was evaluated through comparison to FWHM measurements in fluorescent beads, with the results showing comparable values. For ease of use, an open-source implementation is made available as an ImageJ plugin. This allows application of the method without specialized knowledge of programming or optics. The tool may be of general interest for analysing samples in 3D microscopy where resolution is non-uniform and quantifying image quality at different depths of features is important.

## Supplementary Material

vbad182_Supplementary_Data

## Data Availability

The ImageJ plugin and source code are available from https://www.imperial.ac.uk/rowlands-lab/resources/.
