# Supplementary material for: mtFRC: depth-dependent resolution quantification of image features in 3D fluorescence microscopy
Source: Bioinform Adv. 2023 Dec 18;3(1):vbad182. doi: 10.1093/bioadv/vbad182 (PMC10749749; doi:10.1093/bioadv/vbad182)
Supplement: vbad182_Supplementary_Data [file vbad182_supplementary_data.docx]

# Supplementary Information

## Figure S1: Comparison of Mean Rolling FRC versus Mean Tiled FRC.


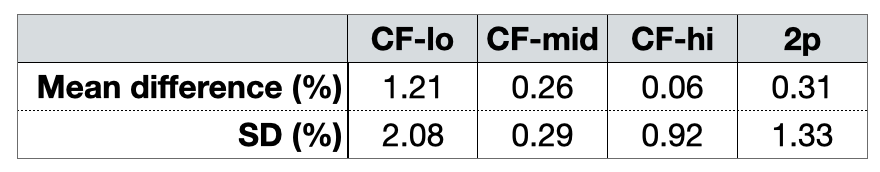


Mean and standard deviation percentage differences for *Mean Rolling FRC* compared to *Mean Tiled FRC* (mtFRC). Results for both methods were computed every 20 μm for the same sample.

## Figure S2: Signal attenuation.


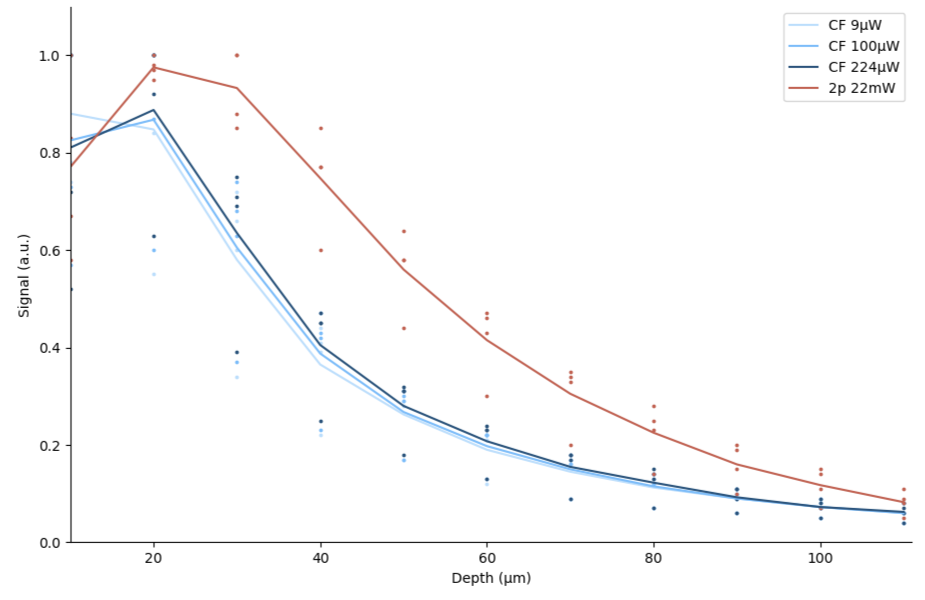


Signal attenuation in nSyb>GFP Drosophila brain using confocal imaging with 3 different power levels (9 μW, 100 μW, 224 μW) and 2-photon imaging at a single power level (22 mW).

Signal attenuation was calculated using the same ROIs used to calculate mtFRC and normalised for each stack to arbitrary units.
